# The efficacy of adjuvant chemotherapy for older adults with stage II/III gastric cancer: a retrospective cohort study

**DOI:** 10.1186/s12885-023-11244-z

**Published:** 2023-08-18

**Authors:** Yu-Hsuan Shih, Hsin-Chen Lin, Po-Wei Liao, Cheng-Wei Chou, Cheng-Hsien Lin, Chiann-Yi Hsu, Chieh-Lin Jerry Teng, Feng-Hsu Wu, Shao-Ciao Luo, Shao-Hsuan Kao

**Affiliations:** 1 Division of Hematology/Medical Oncology, Department of Medicine, Taichung Veterans General Hospital, Taichung, Taiwan; 2 Department of Post-Baccalaureate Medicine, College of Medicine, National Chung Hsing University, Taichung, Taiwan; 3 Institute of Medicine, College of Medicine, Chung Shan Medical University, No. 110, Sec. 1, Jianguo N. Rd, 402 Taichung, Taiwan; 4 Biostatistics Task Force, Taichung Veterans General Hospital, Taichung, Taiwan; 5 Department of Life Science, Tunghai University, Taichung, Taiwan; 6 School of Medicine, Chung Shan Medical University, Taichung, Taiwan; 7 Department of Critical Care Medicine, Taichung Veterans General Hospital, Taichung, Taiwan; 8 Division of General Surgery, Department of Surgery, Taichung Veterans General Hospital, Taichung, Taiwan; 9 Department of Nursing, HungKuang University, Taichung, Taiwan; 10 Department of Medical Research, Chung Shan Medical University Hospital, Taichung, Taiwan

**Keywords:** Older patients, Gastric cancer, Adjuvant chemotherapy, Overall survival, Performance status

## Abstract

**Background:**

Adjuvant chemotherapy is recommended as the standard treatment for patients with stage II/III resected gastric cancer. However, it is unclear whether older patients also benefit from an adjuvant chemotherapy strategy. This study aimed to investigate the clinical impact of adjuvant chemotherapy in older patients with stage II/III gastric cancer.

**Methods:**

This retrospective, real-world study analyzed 404 patients with stage II/III gastric cancer visited at our institute between January 2009 and December 2019. The clinical characteristics and outcomes of patients aged 70 years or older who received adjuvant chemotherapy were compared with those who did not receive this type of treatment. Propensity score analysis was performed to mitigate selection bias.

**Results:**

Of the 404 patients analyzed, 179 were aged 70 years or older. Fewer older patients received adjuvant chemotherapy than did younger patients (60.9% vs. 94.7%, respectively; *P* < 0.001). Among patients aged 70 years or older, those who received adjuvant chemotherapy had improved disease-free survival (DFS) (5-year DFS rate, 53.1% vs. 30.4%; *P* < 0.001) and overall survival (OS) (5-year OS rate, 68.7% vs. 52.1%; *P* = 0.002) compared to those who did not receive adjuvant chemotherapy. A similar survival benefit was observed in the propensity-matched cohort. Multivariate analysis showed that more advanced stage was associated with poorer OS. Receipt of adjuvant chemotherapy was independently associated with a decreased hazard of death (hazard ratio (HR), 0.37; 95% confidence intervals (CI), 0.20–0.68; *P* = 0.002).

**Conclusions:**

Adjuvant chemotherapy may benefit older stage II/III gastric cancer patients aged ≥ 70 years. Further prospective studies are needed to confirm these findings.

**Supplementary Information:**

The online version contains supplementary material available at 10.1186/s12885-023-11244-z.

## Introduction

Gastric cancer is the fifth most common cancer and the fourth leading cause of cancer-related mortality globally, with the highest incidence rates in East Asia [1]. Radical surgery with extended lymph node dissection (D2 lymphadenectomy) is the primary surgical treatment for resectable gastric cancers. Despite this, disease recurrence after surgery is common, occurring in one- to two-thirds of the patients [2].

Postoperative adjuvant chemotherapy is the recommended treatment based on prospective phase III studies, such as the Adjuvant Chemotherapy Trial of S-1 for Gastric Cancer (ACTS-GC) and the Capecitabine and Oxaliplatin Adjuvant Study in Stomach Cancer (CLASSIC). These clinical trials demonstrated that adjuvant chemotherapy improves disease-free survival (DFS) and overall survival (OS) in patients with stage II or III gastric cancer who have undergone D2 gastrectomy [3, 4]. However, most patients enrolled in these adjuvant chemotherapy clinical trials were younger than the real-world population of patients with gastric cancer. In Taiwan, for example, the mean age of patients diagnosed with gastric cancer is 67.2 years, compared to the median age of 63 and 56 years, in the ACTS-GC and CLASSIC studies, respectively [5]. In the ACTS-GC study, only 24% of enrolled patients were older than 70 years, and the OS benefit of adjuvant chemotherapy was not significant in the subgroup of older patients in either study [6].

Older patients tend to have a shorter life expectancy, more comorbidities, and a higher risk of treatment-related complications [7]. Age is an independent prognostic factor for OS in elderly gastric cancer patients, and analysis of the Surveillance, Epidemiology, and End Results (SEER) database showed significant age-based variations in gastric cancer surgery and adjuvant chemotherapy [8, 9]. However, the optimal treatment for older patients remains unclear, with no specific guidelines for older patients with gastric cancers.

In this study, we aimed to investigate the benefit of adjuvant chemotherapy for patients aged 70 years or older with stage II and III gastric cancers in a real-world setting by using a retrospective cohort analysis.

## Patients and methods

### Patients

**Fig. 1:**
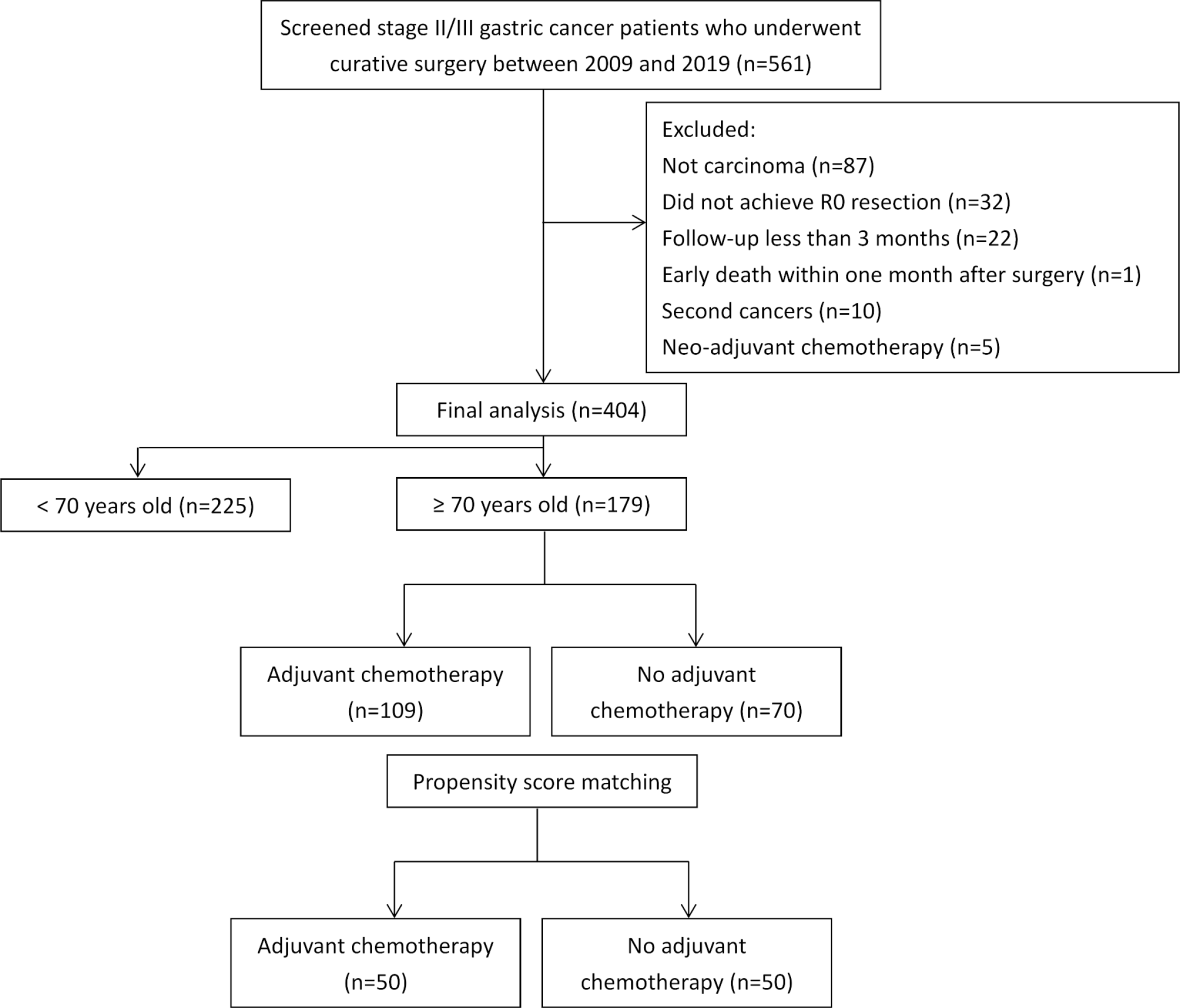
Flow diagram of the study population

We reviewed the medical records of patients diagnosed with postoperative pathological stage II/III gastric cancer between January 2009 and December 2019 at Taichung Veterans General Hospital. We used the eighth edition of the Union for International Cancer Control (UICC) and the American Joint Committee on Cancer (AJCC) staging system to determine the cancer stage. The inclusion criteria for this study were patients aged 20 years and above with histological confirmation of gastric carcinoma and pathological stage II/III, who underwent curative surgery. We excluded patients who did not meet the histology criteria (e.g., histology type other than carcinoma). Patients who did not achieve R0 resection, had a follow-up duration of less than 3 months, received neoadjuvant therapy, had early death within one month after surgery, or had a diagnosis of a second active cancer were also excluded. Of the 561 consecutive cases who met the inclusion criteria, 87 cases were excluded due to non-carcinoma histology, including sarcoma, gastrointestinal stromal tumor, neuroendocrine tumor, neuroendocrine carcinoma, and lymphoma. Additionally, 32 cases were excluded due to positive surgical margin, 22 cases due to a follow-up duration of less than 3 months, 1 case due to early death within 1 month after surgery, 10 cases due to second cancers, and 5 cases due to exposure to neoadjuvant chemotherapy. (Fig. 1)

Of the included 404 patients, 179 were aged 70 years or older. Among the group of older patients, 109 received a minimum of 8 weeks of chemotherapy were included in the chemotherapy group for analysis, while 70 did not. 88% (96 out of 109 patients) received chemotherapy for more than 3 months. The adjuvant chemotherapy regimen consisted of fluoropyrimidines such as S-1, capecitabine, and 5-fluorouracil either alone or in combination with other agents such as cisplatin, oxaliplatin, or taxane, as determined by the physician. The postoperative surveillance strategies for detecting relapse include a history and physical examination every 3–6 months for 5 years, abdominal/pelvic computed tomography every 3–6 months for 5 years, and upper gastrointestinal endoscopy as indicated. This study was conducted in accordance with the principles of the Declaration of Helsinki. The institutional review board of Taichung Veterans General Hospital approved the study and waived the requirement for informed consent due to its retrospective design (No. CE23043C).

### Variables and outcome measurements

The patients’ clinicopathological features were collected retrospectively. We used the age-adjusted Charlson comorbidity index (aCCI) to account for the impact of age on CCI. The variable ‘Year of diagnosis’ is included in the study to assess the potential impact of the progression of time on the survival outcomes of gastric cancer patients. Because in Taiwan, the administration of adjuvant chemotherapy for gastric cancer has evolved over time, with changes such as the inclusion of TS-1 in the national health insurance coverage in 2016. The neutrophil-to-lymphocyte ratio (NLR) was calculated by dividing the neutrophil count (number/𝜇L) by the lymphocyte count (number/𝜇L). The platelet-to-lymphocyte ratio (PLR) was calculated by dividing the platelet count by the lymphocyte count. We used a cut-off value of 2.6 for NLR and 166 for PLR, based on the median value in our cohort. Other measurements included: carcinoembryonic antigen (CEA) and carbohydrate antigen 19 − 9 (CA 19 − 9) levels, and Eastern Cooperative Oncology Group (ECOG) performance status.

The outcomes of this study were DFS and OS. DFS was calculated from the date of diagnosis to the date of disease recurrence or the date of last follow-up. OS was determined from the date of diagnosis to death or the date of last follow-up. The data collection cut-off date was October 31, 2022.

### Statistical analyses

The patients’ clinicopathological characteristics were compared between the groups using the chi-squared test or Fisher’s exact test for categorical variables, and the Mann–Whitney U test for continuous variables. The DFS and OS were analyzed using the Kaplan–Meier method, and the log-rank test was used to assess differences in survival status and recurrence among the groups. We used a Cox proportional hazards model to identify prognostic factors, expressed as hazard ratios (HRs) with 95% confidence intervals (CIs). To reduce treatment selection bias, we performed a propensity score matching analysis. Propensity scores were calculated through logistic regression modeling with the following covariates: age, ECOG performance status, aCCI, year of diagnosis, histologic grade, gastrectomy type, lymphadenectomy type, stage, lymphovascular invasion, perineural invasion, and NLR. These variables were chosen based on their potential impact on the outcome and having a standardized mean difference (SMD) greater than 0.1 between the groups. Patients that received adjuvant chemotherapy and those who did not were matched 1:1 with propensity scores via a nearest neighbor matching algorithm using a caliper size of 0.2 times the logarithm of the standard deviation of the propensity. Standardized differences were estimated before and after matching to evaluate the balance of covariates. Statistical significance was defined as *P* < 0.05. Statistical analyses were performed using the Statistical Product and Service Solutions (IBM SPSS version 22.0; International Business Machines Corp, New York, USA).

## Results

### Comparison of clinical characteristics between younger and older patient groups

Patients in the older age group (≥ 70 years old) had a higher percentage of men (69.3% vs. 52.0%, *P* < 0.001), ECOG performance status > 1 (52.0% vs. 3.1%, *P* < 0.001), aCCI > 3 (77.7% vs. 10.7%, *P* < 0.001), and higher NLR (57.6% vs. 38.5%, *P* < 0.001) compared to the younger age group (< 70 years old; Table 1). Conversely, the signet-ring cell incidence was higher in the younger group of patients. A lower proportion of patients in the ≥ 70 years group received D2 lymphadenectomy (63.7% vs. 80.9%; *P* < 0.001) and adjuvant chemotherapy (60.9% vs. 94.7%; *P* < 0.001) than did patients in the younger group.

**Table 1:** Patient characteristics stratified according to age

	Total (n = 404)		< 70 years (n = 225)		≥ 70 years (n = 179)		*P*-value
**Age, median (IQR)**	67	(57–78)	59	(51–63)	78	(74–83)	< 0.001
**Sex, n (%)**							< 0.001
Female	163	(40.3)	108	(48.0)	55	(30.7)	
Male	241	(59.7)	117	(52.0)	124	(69.3)	
**BMI, n/total n (%)**							0.892
< 18.5	32/400	(8.0)	18/223	(8.1)	14/177	(7.9)	
18.5–23.9	206/400	(51.5)	117/223	(52.5)	89/177	(50.3)	
≥ 24.0	162/400	(40.5)	88/223	(39.5)	74/177	(41.8)	
**ECOG performance status, n (%)**							< 0.001
0–1	304	(75.2)	218	(96.9)	86	(48.0)	
2–4	100	(24.8)	7	(3.1)	93	(52.0)	
**aCCI, n (%)**							< 0.001
0–3	241	(59.7)	201	(89.3)	40	(22.3)	
≥ 4	163	(40.3)	24	(10.7)	139	(77.7)	
**Year of diagnosis, n (%)**							0.547
2009–2013	228	(56.4)	124	(55.1)	104	(58.1)	
2014–2019	176	(43.6)	101	(44.9)	75	(41.9)	
**Histologic grade, n (%)**							0.406
Well or moderately differentiated	61	(15.1)	31	(13.8)	30	(16.8)	
Poorly differentiated	343	(84.9)	194	(86.2)	149	(83.2)	
**Signet-ring feature, n/total n (%)**							< 0.001
No	251/403	(62.3)	114/225	(50.7)	137/178	(77.0)	
Yes	152/403	(37.7)	111/225	(49.3)	41/178	(23.0)	
**Gastrectomy type, n (%)**							0.026
Subtotal gastrectomy	274	(67.8)	163	(72.4)	111	(62.0)	
Total gastrectomy	130	(32.2)	62	(27.6)	68	(38.0)	
**Lymphadenectomy type, n (%)**							< 0.001
< D2 dissection	108	(26.7)	43	(19.1)	65	(36.3)	
D2 dissection	296	(73.3)	182	(80.9)	114	(63.7)	
**Stage, n (%)**							0.843
Stage II	176	(43.6)	99	(44.0)	77	(43.0)	
Stage III	228	(56.4)	126	(56.0)	102	(57.0)	
**Lymphovascular invasion, n/total n (%)**							0.288
No	112/403	(27.8)	67/224	(29.9)	45/179	(25.1)	
Yes	291/403	(72.2)	157/224	(70.1)	134/179	(74.9)	
**Perineural invasion, n/total n (%)**							0.063
No	171/399	(42.9)	86/222	(38.7)	85/177	(48.0)	
Yes	228/399	(57.1)	136/222	(61.3)	92/177	(52.0)	
**Adjuvant chemotherapy, n (%)**							< 0.001
No	82	(20.3)	12	(5.3)	70	(39.1)	
Yes	322	(79.7)	213	(94.7)	109	(60.9)	
**CEA level, n/total n (%)**							0.058
Normal (< 5 U/mL)	328/382	(85.9)	191/215	(88.8)	137/167	(82.0)	
Elevated (≥ 5 U/mL)	54/382	(14.1)	24/215	(11.2)	30/167	(18.0)	
**CA 19 − 9 level, n/total n (%)**							0.217
Normal (< 34 U/mL)	272/330	(82.4)	160/189	(84.7)	112/141	(79.4)	
Elevated (≥ 34 U/mL)	58/330	(17.6)	29/189	(15.3)	29/141	(20.6)	
**NLR, n/total n (%)**							< 0.001
≤ 2.6	211/398	(53.0)	136/221	(61.5)	75/177	(42.4)	
> 2.6	187/398	(47.0)	85/221	(38.5)	102/177	(57.6)	
**PLR, n/total n (%)**							0.266
≤ 166	203/397	(51.1)	118/220	(53.6)	85/177	(48.0)	
> 166	194/397	(48.9)	102/220	(46.4)	92/177	(52.0)	

aCCI, age-adjusted Charlson comorbidity index; BMI, body mass index; CA 19 − 9, carbohydrate antigen 19 − 9; CEA, carcinoembryonic antigen; ECOG, Eastern Cooperative Oncology Group; IQR, interquartile range; NLR, neutrophil-to-lymphocyte ratio; PLR, platelet-to-lymphocyte ratio

### Clinical characteristics in patients aged 70 years or older

Of the patients aged 70 years or older, 109 received adjuvant chemotherapy and 70 did not. Of the chemotherapy group, 90 patients (83%) received a single agent fluoropyrimidine chemotherapy and 19 (17%) received a combination regimen. The chemotherapy regimens used in the 109 patients are listed in Supplementary Table S1. Patients who received adjuvant chemotherapy were younger (77 vs. 81.5, *P* < 0.001), had better ECOG performance statuses (60.6% vs. 28.6% for ECOG performance status 0–1, *P* < 0.001), had lower comorbidity index (28.4% vs. 12.9% for aCCI 0–3, *P* = 0.015), and were more likely to receive D2 lymphadenectomy (69.7% vs. 54.3%, *P* = 0.036) compared to those patients who did not receive adjuvant chemotherapy (Table 2).

**Table 2:** Characteristics of the patients aged 70 years or older, overall cohort and propensity-matched cohort

	Overall cohort						Propensity-matched cohort					
	No chemotherapy (n = 70)		Adjuvant chemotherapy (n = 109)		*P-*value	SMD	No chemotherapy (n = 50)		Adjuvant chemotherapy (n = 50)		*P-*value	SMD
**Age, median (IQR)**	81.5	(76.75-85)	77	(73–81)	< 0.001	0.663	79.5	(75–84)	79.5	(75–83)	0.920	0.004
**Sex, n (%)**					0.620	0.076					0.509	0.133
Female	23	(32.9%)	32	(29.4%)			16	(32.0%)	13	(26.0%)		
Male	47	(67.1%)	77	(70.6%)			34	(68.0%)	37	(74.0%)		
**BMI, n/total n (%)**					0.945	0.051					1.000	< 0.001
< 18.5	6/69	(8.7%)	8/108	(7.4%)			3/49	(6.1%)	3/49	(6.1%)		
18.5–23.9	34/69	(49.3%)	55/108	(50.9%)			23/49	(46.9%)	23/49	(46.9%)		
≥ 24.0	29/69	(42.0%)	45/108	(41.7%)			23/49	(46.9%)	23/49	(46.9%)		
**ECOG performance status, n (%)**					< 0.001	0.680					0.839	0.041
0–1	20	(28.6%)	66	(60.6%)			20	(40.0%)	21	(42.0%)		
2–4	50	(71.4%)	43	(39.4%)			30	(60.0%)	29	(58.0%)		
**aCCI, n (%)**					0.015	0.392					0.603	0.104
0–3	9	(12.9%)	31	(28.4%)			8	(16.0%)	10	(20.0%)		
≥ 4	61	(87.1%)	78	(71.6%)			42	(84.0%)	40	(80.0%)		
**Year of diagnosis, n (%)**					0.255	0.175					1.000	< 0.001
2009–2013	37	(52.9%)	67	(61.5%)			30	(60.0%)	30	(60.0%)		
2014–2017	33	(47.1%)	42	(38.5%)			20	(40.0%)	20	(40.0%)		
**Histologic grade, n (%)**					0.180	0.202					0.444	0.153
well to moderate	15	(21.4%)	15	(13.8%)			11	(22.0%)	8	(16.0%)		
poor differentiation	55	(78.6%)	94	(86.2%)			39	(78.0%)	42	(84.0%)		
**Signet-ring feature, n/total n (%)**					0.682	0.063					0.599	0.106
No	55	(78.6%)	82/108	(75.9%)			39	(78.0%)	36	(73.5%)		
Yes	15	(21.4%)	26/108	(24.1%)			11	(22.0%)	13	(26.5%)		
**Gastrectomy type, n (%)**					0.447	0.116					0.838	0.041
Subtotal gastrectomy	41	(58.6%)	70	(64.2%)			31	(62.0%)	30	(60.0%)		
Total gastrectomy	29	(41.4%)	39	(35.8%)			19	(38.0%)	20	(40.0%)		
**Lymphadenectomy type, n (%)**					0.036	0.322					0.838	0.041
< D2 dissection	32	(45.7%)	33	(30.3%)			19	(38.0%)	30	(60.0%)		
D2 dissection	38	(54.3%)	76	(69.7%)			31	(62.0%)	20	(40.0%)		
**Stage, n (%)**					0.069	0.281					1.000	< 0.001
Stage II	36	(51.4%)	41	(37.6%)			22	(44.0%)	22	(44.0%)		
Stage III	34	(48.6%)	68	(62.4%)			28	(56.0%)	28	(56.0%)		
**Lymphovascular invasion, n/total n (%)**					0.056	0.290					1.000	< 0.001
No	23	(32.9%)	22	(20.2%)			13	(26.0%)	13	(26.0%)		
Yes	47	(67.1%)	87	(79.8%)			37	(74.0%)	37	(74.0%)		
**Perineural invasion, n/total n (%)**					0.463	0.113					0.841	0.040
No	36/70	(51.4%)	49/107	(45.8%)			25	(50.0%)	26	(52.0%)		
Yes	34/70	(48.6%)	58/107	(54.2%)			25	(50.0%)	24	(48.0%)		
**CEA level, n/total n (%)**					0.837	0.033					0.615	0.104
Normal	53/64	(82.8%)	84/103	(81.6%)			39/46	(84.8%)	38/47	(80.9%)		
Elevated (≥ 5 U/ml)	11/64	(17.2%)	19/103	(18.4%)			7/46	(15.2%)	9/47	(19.1%)		
**CA 19 − 9 level, n/total n (%)**					0.832	0.037					0.473	0.106
Normal	41/51	(80.4%)	71/90	(78.9%)			31/38	(81.6%)	33/44	(75.0)		
Elevated (≥ 34 U/ml)	10/51	(19.6%)	19/90	(21.1%)			7/38	(18.4%)	11/44	(25.0)		
**NLR, n/total n (%)**					0.467	0.112					0.841	0.040
≤ 2.6	32/70	(45.7%)	43/107	(40.2%)			23	(46.0%)	22	(44.0%)		
> 2.6	38/70	(54.3%)	64/107	(59.8%)			27	(54.0%)	28	(56.0%)		
**PLR, n/total n (%)**					0.906	0.018					0.689	0.080
≤ 166	34/70	(48.6%)	51/107	(47.7%)			24	(48.0%)	26	(52.0%)		
> 166	36/70	(51.4%)	56/107	(52.3%)			26	(52.0%)	24	(48.0%)		

aCCI, age-adjusted Charlson comorbidity index; BMI, body mass index; CA 19 − 9, carbohydrate antigen 19 − 9; CEA, carcinoembryonic antigen; ECOG, Eastern Cooperative Oncology Group; IQR, interquartile range; NLR, neutrophil-to-lymphocyte ratio; PLR, platelet-to-lymphocyte ratio; SMD, standardized mean differences

### Survival analysis in patients aged 70 years or older

**Fig. 2:**
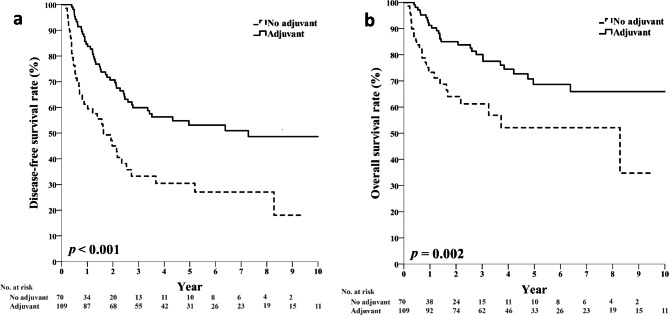
DFS **(a)** and OS **(b)** of patients aged 70 years or older with stage II/III resected gastric cancers, and comparison between patients who received adjuvant chemotherapy and those who did not DFS, disease-free survival; OS, overall survival

Treatment with adjuvant chemotherapy was associated with increased patient DFS and OS, compared to the treatment that did not include adjuvant chemotherapy (Fig. 2). The 5-year DFS and OS rates for the adjuvant chemotherapy group were 53.1% and 68.7%, respectively, compared to 30.4% and 52.1% in the group of patients not treated with chemotherapy (log-rank *P* < 0.001 for DFS, *P* = 0.002 for OS). Subgroup analysis of different disease stages also demonstrated the survival benefit of adjuvant chemotherapy (Supplementary Figure S1, S2). In patients who received adjuvant chemotherapy versus those who did not, the 5-year OS rates were 86.0% vs. 65.6% in the subgroup with stage II, and 57.7% vs. 42.0% in the subgroup with stage III gastric cancer, respectively.

The univariate and multivariate Cox proportional hazards models of OS for patients aged 70 or older are shown in Table 3. In the multivariate analysis, more advanced pathologic stage (stage III HR, 3.96; 95% CI, 2.03–7.73; *P* < 0.001) was associated with poorer OS. Conversely, receipt of adjuvant chemotherapy was independently associated with a decreased hazard of death (HR, 0.37; 95% CI, 0.20–0.68; *P* = 0.002).

**Table 3:** Prognostic factors of patients 70 years and older

	Univariate analysis				Multivariate analysis		
	HR	(95% CI)	*P*-value		HR	(95% CI)	*P*-value
**Adjuvant chemotherapy**							
No	1.00				1.00		
Yes	0.42	(0.24–0.73)	0.002		0.37	(0.20–0.68)	0.002
**Sex**							
Male	1.00				1.00		
Female	0.50	(0.26–0.98)	0.042		0.58	(0.29–1.16)	0.126
**BMI**							
18.5–23.9	1.00						
< 18.5	0.89	(0.27–2.95)	0.854				
≥ 24.0	0.81	(0.45–1.43)	0.457				
**ECOG performance status**							
0–1	1.00				1.00		
2–4	2.39	(1.35–4.24)	0.003		1.79	(0.95–3.36)	0.070
**aCCI**							
0–3	1.00						
≥ 4	1.93	(0.91–4.11)	0.088				
**Year of diagnosis**							
2009–2013	1.00						
2014–2019	1.51	(0.86–2.63)	0.149				
**Histologic grade**							
Grade I/II	1.00						
Grade III	0.98	(0.48–2.02)	0.965				
**Signet-ring feature**							
No	1.00						
Yes	0.81	(0.41–1.61)	0.545				
**Gastrectomy type**							
Subtotal gastrectomy	1.00				1.00		
Total gastrectomy	1.92	(1.11–3.31)	0.019		1.17	(0.65–2.10)	0.600
**Lymphadenectomy type**							
< D2 dissection	1.00				1.00		
D2 dissection	0.53	(0.31–0.92)	0.025		0.74	(0.41–1.33)	0.309
**Stage**							
Stage II	1.00				1.00		
Stage III	3.16	(1.68–5.93)	< 0.001		3.96	(2.03–7.73)	< 0.001
**Lymphovascular invasion**							
No	1.00						
Yes	0.88	(0.48–1.62)	0.677				
**Perineural invasion**							
No	1.00						
Yes	1.72	(0.98–3.03)	0.060				
**CEA level ≥ 5 U/mL**							
No	1.00						
Yes	1.86	(0.96–3.61)	0.064				
**CA 19 − 9 level ≥ 34 U/mL**							
No	1.00						
Yes	1.44	(0.69–3.04)	0.332				
**NLR**							
≤ 2.6	1.00						
> 2.6	1.49	(0.84–2.64)	0.176				
**PLR**							
≤ 166	1.00						
> 166	1.60	(0.91–2.80)	0.103				

aCCI, age-adjusted Charlson comorbidity index; BMI, body mass index; CA 19 − 9, carbohydrate antigen 19 − 9; CEA, carcinoembryonic antigen; CI, confidence interval; ECOG, Eastern Cooperative Oncology Group; HR, hazard ratio; NLR, neutrophil-to-lymphocyte ratio; PLR, platelet-to-lymphocyte ratio

### Clinical characteristics and survival analysis in a propensity-matched cohort of patients aged 70 years or older

**Fig. 3:**
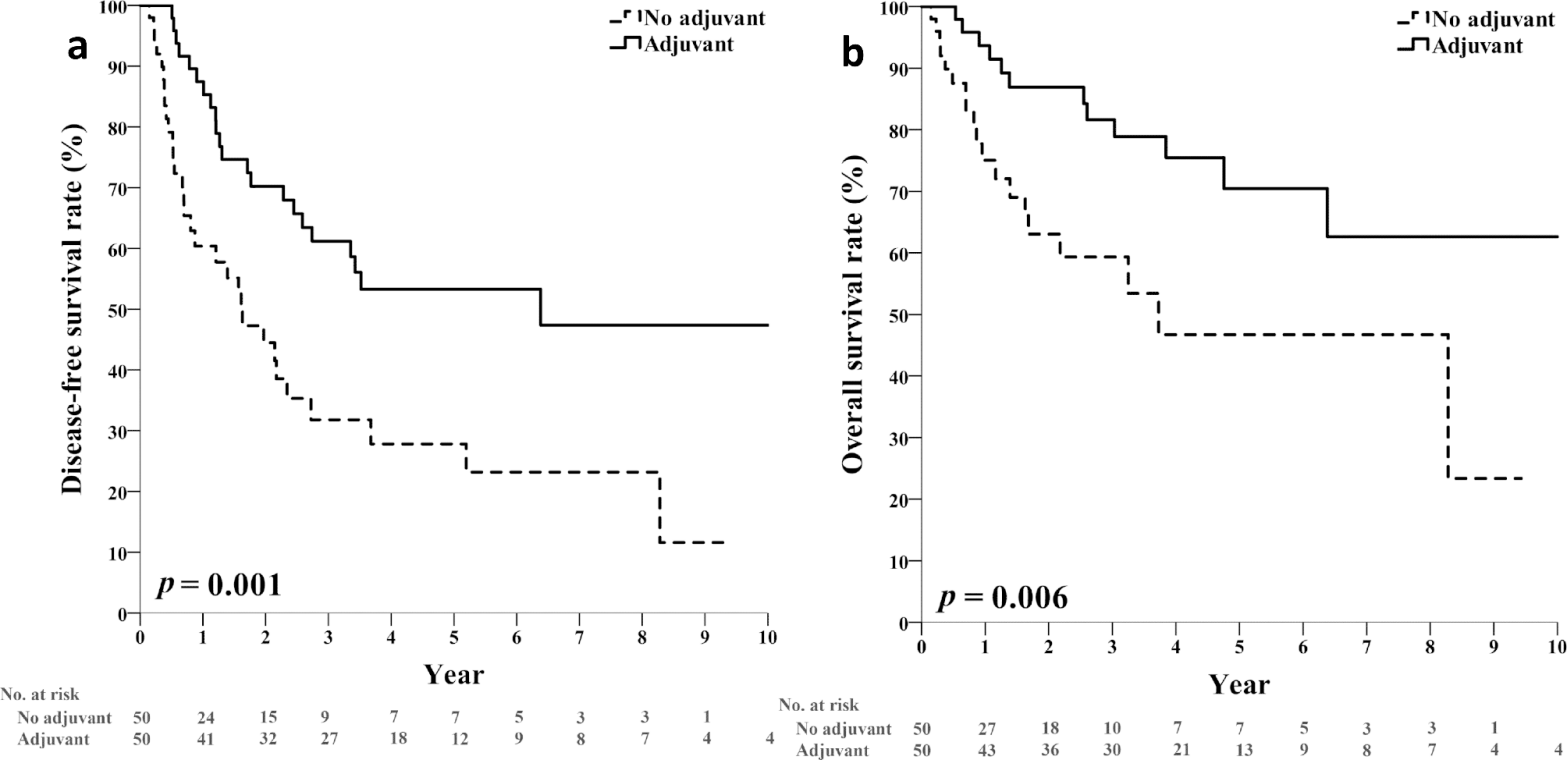
DFS **(a)** and OS **(b)** of patients aged 70 years or older with stage II/III resected gastric cancers, propensity-matched cohort, and comparison between patients who received adjuvant chemotherapy and those who did not DFS, disease-free survival; OS, overall survival

To reduce the influence of bias due to differences in patient’s characteristics or selection, we performed a propensity-matched cohort analysis. In the propensity-matched cohort of 100 patients, 50% received adjuvant chemotherapy. After matching, the previously observed differences between the groups of patients receiving chemotherapy and those who did not have been mitigated. (Table 2). In this cohort, patients who received adjuvant chemotherapy had better 5-year DFS (53.3% vs. 27.8%, log-rank *P* = 0.001) and 5-year OS rates (70.5% vs. 46.7%, log-rank *P* = 0.006) compared to those who did not receive adjuvant chemotherapy (Fig. 3).

### Clinical characteristics and survival analysis in patients aged 70 years or older who underwent D2 surgery

The potential impact of adjuvant chemotherapy might be influenced by whether or not D2 surgery is performed. Therefore, we conducted an analysis excluding patients who did not undergo D2 surgery. The adjuvant chemotherapy group showed significantly higher 5-year disease-free survival (DFS) and overall survival (OS) rates, with rates of 57.9% and 73.3%, respectively, compared to 22.3% and 39.2% in the group of patients who did not receive chemotherapy (log-rank P < 0.001 for DFS, P < 0.001 for OS). The data remained consistent in the propensity-matched cohort (Supplementary Table S3, Figure S3, S4).

## Discussion

Our findings demonstrate that the use of adjuvant chemotherapy is associated with better DFS and OS in patients ≥ 70 years old. In the propensity-matched cohort, we also observed the survival benefit of adjuvant chemotherapy.

Several prospective clinical trials have shown the efficacy of gastrectomy plus chemotherapy for stage II/III gastric cancers. In western countries, the INT-0116 study demonstrated that adjuvant chemoradiotherapy can improve OS [10]. The MAGIC and FLOT4 trials also suggested survival benefits of perioperative chemotherapy [11, 12]. In East Asia, D2 lymphadenectomy followed by adjuvant chemotherapy has been proposed as the standard of care based on the ACTS-GC and CLASSIC studies [3, 13].

In our study, more than 90% of patients in the < 70 years old group received adjuvant chemotherapy, but in the ≥ 70 years old group, only 60.9% of the patients received this type of treatment. This finding is similar to that of previous reports, in which a limited number of older patients received adjuvant chemotherapy [14, 15]. This lower percentage of patients may reflect concerns regarding limited life expectancy, decreased physiologic reserve, frailty, and vulnerability to chemotoxicity in elderly patients. Although clinical guidelines suggest adjuvant chemotherapy for fit patients, [16, 17] physicians and patients might choose a more conservative postoperative strategy after major surgery, in real-world practice.

Older patients are typically underrepresented in clinical trials and thus there is limited information on the optimal treatment course for this group. Several retrospective studies have investigated the efficacy of adjuvant chemotherapy in older patients. One study included 273 patients aged 70 years or older and concluded that postoperative chemotherapy may be unnecessary for elderly patients because it does not provide any survival benefit; however, only 13.2% of the patients (36 of 273) received postoperative chemotherapy [18]. Hoffman et al. used the linked SEER-Medicare database and they also did not detect a survival benefit of adjuvant chemoradiation in elderly patients [19]. In contrast, in a study that included 360 patients aged 65 years or older, 34.7% of patients received fluoropyrimidine-based adjuvant chemotherapy, which provided survival benefits, especially in stage III patients [14]. In another report, a single-center study in Korea analyzed 94 patients aged 70 years or older with stage II or III gastric cancers. The median relapse-free survival of the 55 patients who received adjuvant chemotherapy was 35.5 months, compared with 20.4 months in the 39 patients who received regular checkups only. Ten patients who received adjuvant chemotherapy experienced grade 3 or higher adverse events. The study concluded that adjuvant chemotherapy was associated with longer relapse-free survival and acceptable adverse effects in elderly patients [20]. Recently, Liang et al. conducted a survival analysis of elderly patients over 65 years old with stage II/III gastric cancers. In the 270 patients cohort, adjuvant chemotherapy was significantly associated with higher OS and DFS in stage III patients; only 16 patients experienced grade 3 or higher adverse events [15].

These retrospective studies have inherent limitations; frail and unfit patients may prefer not to undergo chemotherapy, and the survival differences between patients who received chemotherapy or did not receive chemotherapy might be confounded by baseline characteristics. In the present study, we conducted a propensity-matched analysis to balance the baseline differences of age, ECOG performance status, CCI, and D2 lymphadenectomy between groups. The survival benefits of adjuvant chemotherapy in patients aged 70 years or older were noted across the overall and propensity-matched cohorts. Some studies have investigated the efficacy of adjuvant chemotherapy in elderly patients with colon cancer or pancreatic cancer. These studies suggested that receipt of adjuvant chemotherapy was associated with a reduced risk of death, and age alone might not preclude patients from curative management [21, 22]. In our previous study of metastatic gastric cancer, we found that the efficacy of palliative chemotherapy was equivalent between older and younger patients [23].

In our cohort, the disease-free survival (DFS) in the surgery-alone group was found to be lower compared to previous phase III trials, such as the ACTS-GC trial or CLASSIC trial. This disparity can be attributed to several factors specific to our real-world cohort. In the phase III trials, the enrolled patients were typically younger, had better overall fitness, and underwent D2 dissection. In contrast, our cohort consisted exclusively of patients aged 70 years or older, many of whom had an advanced performance status (71.4% had an ECOG PS > 1), and a higher burden of comorbidities (87.1% had an aCCI > 3). Furthermore, only 54.3% of our patients in the surgery-alone group received the standard D2 dissection. These factors could contribute to the significantly lower DFS observed in our surgical group, as depicted in Fig. S1 and S2. This may have influenced the results, indicating a significant difference in the efficacy of adjuvant chemotherapy in this study. Moreover, the potential impact of adjuvant chemotherapy might be influenced by whether or not D2 surgery is performed. Therefore, we conducted an analysis excluding patients who did not undergo D2 surgery. The results of this analysis remained consistent with our findings.

In terms of predicting outcomes for older patients with stage II/III resected gastric adenocarcinoma, we identified adjuvant chemotherapy as an independent positive prognostic factor and more advanced stage was found to be associated with inferior OS; however, D2 lymphadenectomy was not a significant prognostic factor, as shown by multivariate analysis. Gastrectomy with D2 lymphadenectomy has been established as the optimal surgical approach for gastric cancer, based on the 15-year follow-up results of the Dutch trial [24]. Current guidelines recommend D2 lymphadenectomy as the standard surgical procedure; [16, 17] in our cohort, most young patients received D2 lymphadenectomy, but only 63.7% of older patients underwent this procedure. The extent of lymphadenectomy in older patients is still a matter of debate [7, 25]. According to the Dutch trial, patients over the age of 70 had significantly higher morbidity and mortality rates than those aged 70 or lower. The mean OS was 8.69 years for patients aged 70 or lower in the D2 group, whereas it was 5.35 years for patients aged over 70. Rausei et al. reviewed 1,322 patients’ clinical data and found that post-operative complications occurred more frequently in patients over 70 years of age, especially in those with a high Charlson comorbidity score [26]. A retrospective study including 273 patients aged 70 years or older, found that only one-third of patients underwent D2 dissection. The 5-year OS rate did not differ between older patients undergoing D1 or D2 lymph node dissection [18]. In contrast, Brenkman et al. conducted a population-based study and found that a high lymph node yield is associated with prolonged survival in elderly patients [27]. Given these controversial findings, a comprehensive preoperative assessment is necessary to tailor the surgical approach to each individual older patient to avoid under- or over-treatment in this complex patient group.

NLR and PLR are biomarkers associated with cancer-related inflammation. Basic research findings suggest that inflammatory cytokines are associated with adverse biological effects and tumor progression; [28, 29] several studies have shown that high NLR or PLR are poor prognostic factors in gastric cancer [30, 31]. Nevertheless, research on the impact of these biomarkers in older patients is limited. In this study, we compared the inflammatory biomarkers between older and younger patients and found that older patients had higher NLRs. However, multivariate analysis revealed that neither the NLR nor PLR were independent prognostic factors of OS. Further studies are necessary to explore the roles of NLR and PLR in the older patient population.

There were several limitations to our study. First, it was a retrospective study conducted at a single center with a limited patient population, which may affect the generalizability of the findings. Second, there were imbalances in the baseline characteristics between groups. Although we used propensity score analysis to mitigate selection bias, residual confounding factors may still exist. Third, the chemotherapy regimens were not standardized, and there was heterogeneity in the treatments prescribed. In addition, we did not analyze treatment-related adverse effects due to missing data. Despite these limitations, our study provides real-world evidence and contributes to the current understanding of the benefits of adjuvant chemotherapies in older patient groups.

## Conclusions

In conclusion, our findings suggest that adjuvant chemotherapy may confer a survival benefit for patients aged 70 years or older with stage II/III resected gastric cancers. Given that this was a retrospective study, further validation through prospective clinical trials is needed.

### Electronic supplementary material

Below is the link to the electronic supplementary material.

Supplementary Material 1

## Data Availability

The datasets used and/or analyzed during the current study are available from the corresponding author on reasonable request.
