# Supplementary material for: The efficacy of adjuvant chemotherapy for older adults with stage II/III gastric cancer: a retrospective cohort study
Source: BMC Cancer. 2023 Aug 18;23:770. doi: 10.1186/s12885-023-11244-z (PMC10436551; doi:10.1186/s12885-023-11244-z)
Supplement: Supplementary file 1 — Supplementary Material 1 [file 12885_2023_11244_MOESM1_ESM.docx]

**Table S1. Regimens of adjuvant chemotherapy in patients ≥ 70 years old, overall cohort**

| **Chemotherapy Regimens, n (%)** | | N = 109 |
| --- | --- | --- |
| Single-agent regimen, n=90 (82.6%) | Tegafur-uracil (UFT) | 47 (43.1) |
|  | Tegafur-gimeracil-oteracil (S-1) | 36 (33.0) |
|  | 5-Flurouracil | 7 (6.4) |
| Combination regimen, n=19 (17.4%) | Capecitabine + Oxaliplatin | 16 (14.7) |
|  | Oxaliplatin + 5-Flurouracil | 2 (1.8) |
|  | Paclitaxel + 5-Flurouracil | 1 (0.9) |

**Table S2. Regimens of adjuvant chemotherapy in patients ≥ 70 years old, propensity-matched cohort**

| **Chemotherapy Regimens, n (%)** | | N = 50 |
| --- | --- | --- |
| Single-agent regimen, n=43 (86.0%) | Tegafur-uracil (UFT) | 24 (48.0) |
|  | Tegafur-gimeracil-oteracil (S-1) | 17 (34.0) |
|  | 5-Flurouracil | 2 (4.0) |
| Combination regimen, n=7 (14.0%) | Capecitabine + Oxaliplatin | 6 (12.0) |
|  | Oxaliplatin + 5-Flurouracil | 1 (2.0) |

| **a** | **b** |
| --- | --- |
|  |  |

**Fig. S1**

DFS (a) and OS (b) of patients aged 70 years or older with stage II resected gastric cancers, and comparison between patients who received adjuvant chemotherapy and those who did not.

DFS, disease-free survival; OS, overall survival

| **a** | **b** |
| --- | --- |
|  |  |

**Fig. S2**

DFS (a) and OS (b) of patients aged 70 years or older with stage III resected gastric cancers, and comparison between patients who received adjuvant chemotherapy and those who did not.

DFS, disease-free survival; OS, overall survival

**Table S3. Characteristics of the patients aged 70 years or older who underwent D2 surgery, overall cohort and propensity-matched cohort.**

|  | **Overall cohort** | | | | | | **Propensity-matched cohort** | | | | | |
| --- | --- | --- | --- | --- | --- | --- | --- | --- | --- | --- | --- | --- |
|  | **No  chemotherapy  (n = 38)** | | **Adjuvant  chemotherapy  (n = 76)** | | ***P-*value** | **SMD** | **No  chemotherapy  (n = 28)** | | **Adjuvant chemotherapy  (n = 28)** | | ***P-*value** | **SMD** |
| **Age, median (IQR)** | 80.5 | (74.75-84) | 75 | (73-81) | 0.003 | 0.660 | 78 | (73.25-81) | 78 | (73.25-82.75) | 0.621 | 0.122 |
| **Sex, n (%)** |  |  |  |  | 0.393 | 0.169 |  |  |  |  | 1.000 | <0.001 |
| Female | 14 | (36.8%) | 22 | (28.9%) |  |  | 9 | (32.1%) | 9 | (32.1%) |  |  |
| Male | 24 | (63.2%) | 54 | (71.1%) |  |  | 19 | (67.9%) | 19 | (67.9%) |  |  |
| **BMI, n/total n (%)** |  |  |  |  | 0.141 | 0.365 |  |  |  |  | 0.216 | 0.525 |
| < 18.5 | 3 | (7.9%) | 1 | (1.3%) |  |  | 3 | (19.7%) | 0 | (0.0%) |  |  |
| 18.5–23.9 | 16 | (42.1%) | 41 | (53.9%) |  |  | 12 | (42.9%) | 16 | (57.1%) |  |  |
| ≥ 24.0 | 19 | (50.0%) | 34 | (44.7%) |  |  | 13 | (46.4%) | 12 | (42.9%) |  |  |
| **ECOG performance status, n (%)** |  |  |  |  | 0.033 | 0.431 |  |  |  |  | 1.000 | <0.001 |
| 0–1 | 16 | (42.1%) | 48 | (63.2%) |  |  | 16 | (57.1%) | 16 | (57.1%) |  |  |
| 2–4 | 22 | (57.9%) | 28 | (36.8%) |  |  | 12 | (42.9%) | 12 | (42.9%) |  |  |
| **aCCI, n (%)** |  |  |  |  | 0.024 | 0.482 |  |  |  |  | 1.000 | 0.097 |
| 0-3 | 5 | (13.2%) | 25 | (32.9%) |  |  | 5 | (17.9%) | 4 | (14.3%) |  |  |
| ≥ 4 | 33 | (86.8%) | 51 | (67.1%) |  |  | 23 | (82.1%) | 24 | (85.7%) |  |  |
| **Year of diagnosis, n (%)** |  |  |  |  | 0.133 | 0.299 |  |  |  |  | 0.783 | 0.074 |
| 2009-2013 | 20 | (52.6%) | 51 | (67.1%) |  |  | 18 | (64.3%) | 17 | (60.7%) |  |  |
| 2014-2017 | 18 | (47.4%) | 25 | (32.9%) |  |  | 10 | (35.7%) | 11 | (39.3%) |  |  |
| **Histologic grade, n (%)** |  |  |  |  | 0.124 | 0.297 |  |  |  |  | 0.313 | 0.272 |
| well to moderate | 10 | (26.3%) | 11 | (14.5%) |  |  | 7 | (25.0%) | 4 | (14.3%) |  |  |
| poor differentiation | 28 | (73.7%) | 65 | (85.5%) |  |  | 21 | (75.0%) | 24 | (85.7%) |  |  |
| **Signet-ring feature, n/total n (%)** |  |  |  |  | 0.848 | 0.038 |  |  |  |  | 0.217 | 0.339 |
| No | 29 | (76.3%) | 56/75 | (74.7%) |  |  | 20 | (71.4%) | 23/27 | (85.2%) |  |  |
| Yes | 9 | (23.7%) | 19/75 | (25.3%) |  |  | 8 | (28.6%) | 4/27 | (14.8%) |  |  |
| **Gastrectomy type, n (%)** |  |  |  |  | 0.886 | 0.028 |  |  |  |  | 0.577 | 0.149 |
| Subtotal gastrectomy | 26 | (68.4%) | 53 | (69.7%) |  |  | 19 | (67.9%) | 17 | (60.7%) |  |  |
| Total gastrectomy | 12 | (31.6%) | 23 | (30.3%) |  |  | 9 | (32.1%) | 11 | (39.3%) |  |  |
| **Stage, n (%)** |  |  |  |  | 0.424 | 0.159 |  |  |  |  | 1.000 | <0.001 |
| Stage II | 19 | (50.0%) | 32 | (42.1%) |  |  | 15 | (53.6%) | 15 | (53.6%) |  |  |
| Stage III | 19 | (50.0%) | 44 | (57.9%) |  |  | 13 | (46.4%) | 13 | (46.4%) |  |  |
| **Lymphovascular invasion, n/total n (%)** |  |  |  |  | 0.037 | 0.409 |  |  |  |  | 0.383 | 0.235 |
| No | 15 | (39.5%) | 16 | (21.1%) |  |  | 10 | (35.7%) | 7 | (25.0%) |  |  |
| Yes | 23 | (60.5%) | 60 | (78.9%) |  |  | 18 | (64.3%) | 21 | (75.0%) |  |  |
| **Perineural invasion, n/total n (%)** |  |  |  |  | 0.063 | 0.380 |  |  |  |  | 0.350 | 0.254 |
| No | 25 | (65.8%) | 35/74 | (47.3%) |  |  | 18 | (64.3%) | 14/27 | (51.9%) |  |  |
| Yes | 13 | (34.2%) | 39/74 | (52.7%) |  |  | 10 | (35.7%) | 13/27 | (48.1%) |  |  |
| **CEA level, n/total n (%)** |  |  |  |  | 0.746 | 0.066 |  |  |  |  | 0.734 | 0.094 |
| Normal | 30/36 | (83.3%) | 60/70 | (85.7%) |  |  | 20/26 | (76.9%) | 21/26 | (80.8%) |  |  |
| Elevated (≥ 5 U/ml) | 6/36 | (16.7%) | 10/70 | (14.3%) |  |  | 6/26 | (23.1%) | 5/26 | (19.2%) |  |  |
| **CA 19-9 level, n/total n (%)** |  |  |  |  | 0.798 | 0.055 |  |  |  |  | 1.000 | 0.107 |
| Normal | 28/33 | (84.8%) | 53/64 | (82.8%) |  |  | 19/24 | (79.2%) | 20/24 | (83.3) |  |  |
| Elevated (≥ 34 U/ml) | 5/33 | (15.2%) | 11/64 | (17.2%) |  |  | 5/24 | (20.8%) | 4/24 | (16.7) |  |  |
| **NLR, n/total n (%)** |  |  |  |  | 0.350 | 0.187 |  |  |  |  | 0.181 | 0.363 |
| ≤ 2.6 | 21 | (55.3%) | 34/74 | (45.9%) |  |  | 16 | (57.1%) | 11 | (39.3%) |  |  |
| > 2.6 | 17 | (44.7%) | 40/74 | (54.1%) |  |  | 12 | (42.9%) | 17 | (60.7%) |  |  |
| **PLR, n/total n (%)** |  |  |  |  | 0.690 | 0.080 |  |  |  |  | 0.786 | 0.073 |
| ≤ 166 | 20 | (52.6%) | 36/74 | (48.6%) |  |  | 16 | (57.1%) | 17 | (60.7%) |  |  |
| > 166 | 18 | (47.4%) | 38/74 | (51.4%) |  |  | 12 | (42.9%) | 11 | (39.3%) |  |  |

aCCI, age-adjusted Charlson comorbidity index; BMI, body mass index; CA 19-9, carbohydrate antigen 19-9; CEA, carcinoembryonic antigen; ECOG, Eastern Cooperative Oncology Group; IQR, interquartile range; NLR, neutrophil-to-lymphocyte ratio; PLR, platelet-to-lymphocyte ratio; SMD, standardized mean differences.

| **a** | **b** |
| --- | --- |
|  |  |

**Fig. S3**

DFS (a) and OS (b) of patients aged 70 years or older with stage II/III gastric cancers who underwent D2 surgery, and comparison between patients who received adjuvant chemotherapy and those who did not.

DFS, disease-free survival; OS, overall survival

| **a** | **b** |
| --- | --- |
|  |  |

**Fig. S4**

DFS (a) and OS (b) of patients aged 70 years or older with stage II/III gastric cancers who underwent D2 surgery, propensity-matched cohort, and comparison between patients who received adjuvant chemotherapy and those who did not.

DFS, disease-free survival; OS, overall survival
